# AIT 2023: Current innovation and future outlook 

**DOI:** 10.5414/ALX02379E

**Published:** 2023-12-12

**Authors:** Magdalena Zemelka-Wiacek, Marek Jutel

**Affiliations:** 1 Department of Clinical Immunology, Wroclaw Medical University, Wroclaw, and; 2 ALL-MED Medical Research Institute, Wroclaw, Poland

**Keywords:** allergen immunotherapy (AIT), biomarkers, biologicals, component-resolved diagnosis (CRD), randomized controlled trial (RCT), real-world evidence (RWE)

## Abstract

Although used for over 100 years, allergen immunotherapy (AIT) is still an indispensable tool in modern allergy managemen20t due to its potential to cure allergic diseases. Its current rapid development through the application of personalized and precision medicine approaches is strongly supported by advances in mHealth, component-resolved diagnosis (CRD)-based diagnostics, validation of novel biomarkers, advanced data management, and development of novel preparations. This review summarizes the key advances in the field and shows the perspectives for further development of next-generation AIT treatments.

## Introduction 

Allergen immunotherapy (AIT) has been widely recognized as an effective and safe treatment for allergic rhinitis and asthma. The major advantages compared to pharmacotherapy include long-term relief of symptoms, prevention of progression from allergic rhinitis to asthma especially in children, and the potential for preventing sensitization in at-risk individuals [1, 2, 3]. This can be achieved by modulation of both the innate and adaptive immune responses associated with consecutive activation of the mechanisms of desensitization, and intermediate- and long term-tolerance, which persists for years after cessation of the AIT treatment. Defining theratypes, which identify therapy responders and non-responders based on the compilation of pathophysiological mechanisms explaining disease expression and response to therapy in different groups of patients, are of major help. 

In this review, we present the current advances in AIT and assess the perspectives of the key pathways of its future development. This includes better defining patients’ theratypes, novel more effective patient enrollment strategies, application of prospective and retrospective biomarkers, a new methodology for clinical research including adaptive study design and advanced data management as well as the development of innovative preparations. 

## Recent advances in understanding the mechanisms of AIT 

**Figure 1.**
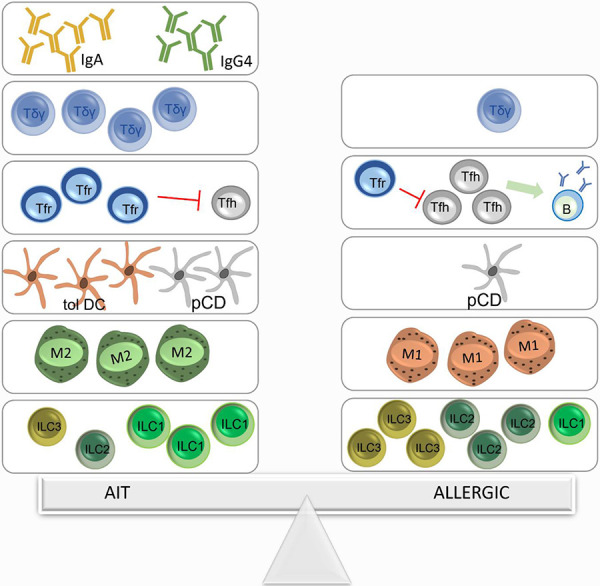
Key advances in the understanding of AIT mechanisms. Tfh cells have a central role in regulating T2 inflammation and IgE secretion, which can be limited by Tfr cells. During AIT, ILC2 and ILC3 decrease while ILC1 increases, resulting in a ratio similar to healthy subjects. A newly identified ILC subgroup that produces IL-10 was found to rise following AIT and correlate with symptom reduction. A proinflammatory monocyte turns its phenotype toward an anti-inflammatory cell, and plasmacytoid dendritic cells augment. Memory and naïve γδTreg cells support the tolerogenic effect. Local specific IgA and serum-IgG4 Ab are upregulated. Under the influence of the allergoid-mannan conjugates, monocytes from healthy and allergic individuals can be reprogrammed into tol DC cells. Ab = antibody; AIT = allergen immunotherapy; B = lymphocytes B cell; IgE/G_4_/A = immunoglobulin type E/G_4_/A; ILC1/2/3 = innate lymphoid cells type 1/2/3; M1/M2 = proinflammatory (nonclassical)/anti-inflammatory (intermediate) monocytes; pDC = plasmacytoid dendritic cells; T = lymphocyte cell; Tfh = follicular T helper cells; Tfr = T follicular regulatory cell; tol DC = tolerogenic dendritic cells; Tγδ = naive and memory gamma delta T regulatory cells.

Key advances in the understanding of AIT mechanisms are presented in Figure 1. 

Recent studies indicate that it is not only the shift from type 2 T-helper lymphocytes (T2) to type 1 (T1) immune response but also the decreased frequencies of follicular helper (Tfh) cells that play the essential role in the induction of tolerance during successful AIT with the consecutive decrease in immunoglobulin (Ig) E synthesis. Anti-inflammatory and regulatory interleukin (IL)-10 secreting by follicular regulatory T (Tfr) cells, a subset of Treg cells, which impairs the action of Tfh are essential in this process [4]. In allergic individuals, the imbalance between these populations is observed, with an increase in IL-13-producing Tfh and a decrease in Tfr cells. AIT can renew this ratio [5]. In addition, in allergic patients, circulating CXCR5^+^PD-1^+^ follicular helper T (cTfh) cells, which secrete IL-4 and IL-21, have been shown to increase IgE production. When comparing groups of patients receiving subcutaneous immunotherapy (SCIT) or sublingual immunotherapy (SLIT), a lower amount of IL-4 and IL-21 was observed in the nasal discharge of these individuals compared to untreated subjects. Additionally, a shift in the chromatin landscape in cTfr and Tfr cells was noted in both the SCIT and SLIT groups. 

B regulatory lymphocytes (Breg) cells contribute to the formation and maintenance of immunological tolerance during AIT by suppressing effector T cells, inhibiting dendritic cell maturation, inducing Treg cells, and producing anti-inflammatory IgG4 antibodies [6]. The assessment of nasal and systemic antibody (Ab) levels during SLIT and SCIT with grass pollen allergens revealed an increase in local and systemic IgA1/2 in SLIT, but serum IgG was higher in SCIT [7]. In the case of a new type of AIT preparation, i.e., allergoid-mannan conjugates that affect dendritic cells (DCs) and generate Treg cells, it was reported that monocytes can be reprogrammed into IL-10^+^ tolerogenic dendritic cells (tolDC), which enrich the expression of anti-inflammatory factors [8]. 

In pediatric patients after peanut oral immunotherapy (POIT), the expression of naive and memory gamma delta T regulatory (γδTreg) cells displayed levels and gene expression (performed by single-cell RNA sequencing) comparable to healthy controls. AIT changed the repertoire of innate cells to resemble that of healthy individuals. Monocytes changed from proinflammatory to anti-inflammatory, the frequency of CD25^+^ innate lymphoid cells (ILCs), plasmacytoid dendritic cells (pDCs), and CD141^+^ myeloid dendritic cells (mDCs) increased, but ILC2s and ILC3c cells decreased. The IL-10^+^-cytotoxic T lymphocyte-associated protein 4^+^ (CTLA-4) ILCs increased after AIT in responders compared to non-responders or the placebo group. There was also a correlation between symptom improvement and the percentage of IL-10^+^ CTLA-4^+^ ILCs [9]. 

In a murine model, oral immunotherapy (OIT) resulted in the production of IL-10^+^ CD4^+^ cells, IL-4^+^ and IL-10^+^ Th2 cells, and myeloid-derived suppression cells. These cells collaborated to induce sustained unresponsiveness (SU) to food allergens, and Notch signaling contributed to the development of these cells. Notch signaling is a crucial biological pathway that influences the development and lineage decisions of various immune cells, including thymic T cells, marginal zone B cells, or splenic CD8 dendritic cells, ultimately leading to the transcriptional expression of target genes. Also, in the murine model of epicutaneous immunotherapy (EPIT), skin dendritic cells (skDCs) like Langerhans cells (LCs) and conventional dermal cDC1 and cDC2 subsets cells acquired varied phenotypic and functional specializations. LCs lost their ability to stimulate CD4^+^ Teff but acquired skills to stimulate Treg; on the other hand, cDC1 lost the capacity to prime CD4^+^ Teff, while cDC2 gained the capacity to induce Th1/17 responses [10]. 

## Novel patient enrollment strategies 

Precision medicine is a prerequisite of successful AIT. The key elements include precise definition of disease phenotype and endotype, application of novel diagnostic tools (component-resolved diagnosis (CRD)), in order to apply the targeted therapy. In a broader sense, the personalized approach should also include the adjustment to the personal and human (personomics, humanomics) background of each patient. This ensures optimal treatment efficacy [11]. 

### Novel biomarkers and clustering 

New biomarkers for patient stratification are shown in Table 1. 

Serum level of periostin, a protein involved in the pathophysiology of allergic rhinitis, has been associated with the effectiveness of SLIT by predicting clinical response. Patients with allergic asthma during the bronchial challenge who developed both, the early (EAR) and late asthmatic reaction (LAR), with additional elevated eosinophilic inflammation, are more likely to respond to AIT [12]. 

In SCIT therapy for grass pollen, the reduction in basophil sensitiveness after 3 weeks of SCIT indicated long-term improvement of symptoms. A decrease in basophil sensitivity was observed during the 1^st^ year of treatment [13]. 

After 24 months of AIT in house dust mite (HDM)-triggered allergic rhinitis responders, the number of CTLA-4^+^ IL-10^+^ ILCs was increased. Using a clustering tool, it was shown that the mechanism was based on the modification of the retinol metabolic pathway, cytokine-cytokine receptor interaction, and Janus kinase signal transducer and activator of transcription protein (JAK-STAT) signaling. Hierarchical clustering of grass pollen-specific serum IgA, IgE, and IgG4 was able to differentiate between placebo and SCIT/SLIT, measuring from baseline to 1 year of treatment. IgA1 quantity in the nasal fluid was associated with a decrease in nasal symptoms during SLIT [9]. Metabolic biomarkers, like arachidonic acid and its downstream metabolites (5(S)-HETE (hydroxyeicosatetraenoic acid), 8(S)-HETE, 11(S)-HETE, 15(S)-HETE, and 11-dehydro thromboxane B2), showed reduction after ~ 1 year of SCIT treatment, and the changes were associated with the clinical symptoms [14]. 

AIT failure in allergic rhinitis patients was correlated with the presence of CD38^+^ B lymphocytes that secrete IL-6 and consequently transform Treg cells into Th17 cells. In addition, the number of B cells was associated with a decreased frequency of Tfr. In OIT with baked egg or peanut, it was found that high levels of allergen-specific IL-4^+^ and IL-13^+^ CD4^+^ cells (T2 immune response) correlated with OIT failure [10, 15]. 

### Component-resolved diagnosis 

The use of whole allergen extracts in allergy diagnosis might be associated with some negative aspects. This can be overcome through CRD, an approach utilized to characterize the molecular components of each allergen involved in a specific IgE (sIgE)-mediated response [16]. CRD can support estimating the chance of success of AIT, especially in the case of polysensitization. CRD is particularly helpful concerning sensitization to panallergens, components that explain positive skin prick tests (SPT), but sensitization to panallergens alone is no indication for AIT. Another important advantage of CRD is the differentiation between true sensitization and cross-reactivity to allergen components [17]. In addition, low sensitization to major allergen components might result in a poorer therapeutic effect in grass pollen AIT. It has been shown that sensitization to Phl p 1 and 5 is essential, and at least one is needed for AIT efficacy [18]. 

Clinical decision support systems have been developed which incorporate clinical history, diagnostic tests including CRD, and eDiaries. They significantly enhance the diagnostic precision and assure the precision approach. An algorithm, based on a pyramid model has been postulated (a clinical decision support system @IT2020-CDSS) [19]. 

## Advanced data management 

Big data in biomedicine can be divided into two broad types of data: omics and non-omics data. Non-omics data include laboratory tests, imaging or morphologic parameters, environment biomonitoring, mobile health (mHealth) records, and clinical registries by healthcare specialists. Omics data are acquired using high-throughput biological platforms and deliver thousands of features characterizing biological processes at different levels. Omics data include genomics, epigenomics, metabolomics, transcriptomics, and proteomics. The majority of studies use a single omic technique to describe biological features. The strength of omics investigation will significantly expand if we can integrate them to develop a comprehensive molecular profile of what is occurring in a specific sample. Precision medicine aims to stratify patients into clusters characterized by common clinical and biological features. Both non-omics and omics data are effective instruments in the interpretation of allergic diseases. A combination of clinical and high-throughput data assembles new possibilities to understand genotype-phenotype relations in allergic subjects by enhancing healthcare workflow, efficient diagnostics, and treatment [20]. 

Artificial intelligence (AI) is an emerging area that leverages effective computer algorithms to solve difficult tasks that normally require human-level intelligence to accomplish. A sub-field within AI known as machine learning (ML) involves using algorithms specially created to ingest large quantities of complex data and to automatically and unbiasedly extract meaning and insights. AI/ML is already used for monitoring airborne pollen count, air pollution risk prediction (long-term exposure to air pollution contributes to exacerbating respiratory diseases), predicting disease sub-phenotypes in patients with food allergies, for the stratification of patients into distinct disease or exposure subgroups, risk stratifications, cluster analysis, and identification of biomarkers. AI can also contribute to increasing efficiency of immunotherapy, reducing healthcare costs, or improving patients’ quality of life. Real-world data is collected in huge quantities in passive ways, such as in hospital systems or in routine public health data collection procedures. Nevertheless, these kinds of datasets are less structured, e.g., a lot of information is locked away in free text, there are many data quality issues to be solved, and there are missing data fields. These problems impeded traditional analysis, but there are ways that AI is beginning to help unlock the potential within these datasets [21]. 

## Applications of mobile health 

mHealth seems to be fundamental to the future of allergy management. mHealth biomarkers enable monitoring of disease status, for example, by online assessment of symptom-medication scores that allow stratification to AIT and follow-up during AIT. The monitoring of efficacy, safety, and medication reduction allows for the identification of clinical responders and non-responders. mHealth technologies could be implemented through electronic reminder systems, e‐communication channels, the use of “push” messaging, gaming, and social networks. Mobile Airways Sentinel Network (MASK) is an example of an application freely available in 27 countries where users self-assess their symptoms and medication usage. The MASK algorithm, based on the visual analog scale (VAS) has unique data on pharmacotherapy that can be used in real-world evidence (RWE). The MASK-air European study found that most patients do not comply with the physicians’ prescriptions and treat themselves according to the severity of their symptoms. Another example is an electronic personal health environment (ePHE), which could be useful for the transition of AIT to primary care, because most patients prefer to have medical care nearby. 

## New methodology for clinical research 

### Real world evidence 

Real-word data (RWD) and the resulting RWE provide supportive data in areas where data from clinical trials are missing or are very rare. Currently, RWE is included in applications to the regulatory authorities. RWE can assess clinical outcomes for unselected populations, potentially help to translate findings from randomized controlled trials (RCTs) into evidence that can guide routine clinical practice. Both RCTs and RWE studies provide endpoints concerning effectiveness and safety of AIT. The main limitation of RCTs in AIT is their short duration, usually no more than a year, but this can be supplemented by RWE. The results from these two sources are assumed to be complementary, but whether they are comparable remains unknown [22]. Based on the knowledge from RWE and the perspectives and recommendations of authorities and scientific societies, there should be a hierarchy of RWE in AIT that places pragmatic trials and registry data at the highest levels of proof. 

In some cases, a large clinical study with pre-planned subgroup analyses is not sufficient to understand the different treatment outcomes. With the growing understanding of the complexity underlying patient heterogeneity, even the most extensive studies will not consider all known subpopulations [23]. The efficacy of AIT has been evaluated in several RWE investigations using large databases. The investigation of the results of the RWE studies confirms the long-term efficacy of AIT demonstrated in RCTs. Recent studies under RWE conditions show significant and also age-dependent differences between adherence to SCIT and SLIT products. There is a need for data on the credibility of RWE in allergic respiratory diseases [24]. The RWE articles published in the field of allergy have increased, except for AIT; there were only 24 papers in 2020. In AIT studies, it is challenging to combine global retrospective data sources due to differences in treatment methods and prescribed products. Proxies for outcomes are often used in retrospective studies, like use of corresponding medications or diagnosis, instead of disease severity based on symptom score. EAACI started a systematic review of observational studies in AIT by implementing the Real-Life Evidence Assessment Tool (RELEVANT) and the Grading of Recommendations Assessment, Development and Evaluation approach (GRADE) to evaluate the quality of the evidence base as a whole [25]. 

There is a need to create more AIT registries that manage data in a consistent manner using standard protocols. This will be the basis of real data that will enable evidence-based study and advancement in research strategy and clinical decision-making. The most recent data from registries include the BRIT (British Society for Allergy and Clinical Immunology – BSACI Registry for ImmunoTherapy) to provide real-life data on safety and efficacy parameters for AIT and biologicals for allergies and urticaria [26], the OmaBASE registry (an online database that collected data from patients with food allergy receiving AIT) [27], or the Danish National Health Service Prescription Database and Statistics Denmark Database [28]. 

In conclusion, the RCTs have to adapt and evolve to provide meaningful data to respond to a changing environment so that the quality of the trial structure, layout, and also effectiveness of generating clinical understanding can be improved. The main issues derive from globalization, external validation, study populations, precision medicine, and clinical trial clarity [23]. RWE provides an important complementary element to solve these problems. 

### Adaptive study design – field vs. chamber studies 

Allergen exposure chambers (AECs) are the real-world model of allergic rhinitis, which is of use for confirming allergy diagnosis or as a plausible endpoint in clinical trials as well as for monitoring safety and efficacy of subcutaneous and sublingual AIT in terms of new formulations or dosing regiments. They present a reasonable method for the stratification of patients into potential responders and non-responders. Challenges in the AEC ensure stable and continuous allergen concentrations and atmosphere, with reproducible results. Chambers can be valuable in long-term investigations when it is not possible to assess natural exposure due to low allergen counts [29]. The validation of individual AEC facilities as well as the demonstration of the correlation between field and AEC exposure are currently the major issues [30]. 

## Assessment of cost-effectiveness 

Assessing the cost-effectiveness of AIT is much needed for clinical decision-making, such as the reimbursement of new interventions. A study on the cost-effectiveness of SCIT and SLIT in pediatric patients with HDM-triggered allergic asthma was conducted in Portuguese subjects. Both strategies were cost-effective, especially SCIT, which reduced medication use and exacerbations leading to emergency unit visits. Overall, SLIT had a greater impact on quality of life [31]. Another study showed that in HDM-induced asthma, SCIT in combination with inhaled corticosteroids (ICS) was cost-effective compared to ICS alone [32]. 

## Advances in AIT compounds 

Current state of the art in AIT compounds is presented in Table 2. 

### Novel preparations 

Clinical trials show that recombinant allergen-based preparations are effective in the treatment of grass and birch pollen allergy [33]. Such constructs can include wild-type allergens or hypoallergenic variants or derivatives including peptides [34]. 

Clinical studies of preparations produced using molecular methods showed much higher levels of induced IgG4 blocking antibodies compared to standard extracts [35]. It was found that SLIT with recombinant Bet v 1 induces individual repertoires of cross-reactive IgG1 and IgG4 antibodies, which display highly variable cross-blocking activity to different allergens [36]. Recently, a passive immunization by recombinant IgG monoclonal antibody (mAb) directed against specific epitopes of allergens has been investigated. These preparations can induce effective symptom reduction, particularly in allergic patients with clinically relevant (mono)sensitization to a particular major allergen, as exemplified using Fel d 1-sensitized cat-allergic patients [37]. 

A number of novel adjuvants with immunomodulatory and release-slowing properties have been investigated. Biocompatible and biodegradable/non-biodegradable nanoparticles may act as adjuvants and may also protect allergens from enzymatic digestion. It is possible that the nanoparticles may be complexes that are loaded with allergens (research is carried out in vitro and on animals). Unspecific immune stimulators like polysaccharide polymers, liposomes, glycodendrimers, or virus-like particles (VLPs) also are being tested as new AIT compounds [38]. 

Glutaraldehyde-polymerized allergoids conjugated to non-oxidized mannan (allergoid-mannan conjugates) are next-generation AIT preparations targeting DCs. Allergoid-mannan conjugates are captured by DCs through an internalization mechanism, subsequently induce blocking antibodies, and promote the generation of forkhead box 3 (FOXP3)-positive Treg cells; they also reprogram monocytes into stable tolerogenic DCs [8]. 

### Biologicals and AIT 

The strategy of combining AIT with biological treatment has been investigated over the last decades. The application of omalizumab, an anti-IgE mAb, has been investigated. Omalizumab as OIT add-on in severe food-allergic subjects effectively lowered the risk of allergic side effects; however, the effect was short-lasting and did not result in increased efficacy [39]. This has been confirmed in other studies in respiratory, food, or insect venom allergy. Similar observations have been made in a more recent study on dupilumab in allergic rhinitis subjects [40]. Tezepelumab, a human mAb anti-thymic stromal lymphopoietin (TSLP), enhances the efficacy of SCIT in cat-triggered allergic rhinitis patients and may promote tolerance after a 1-year course of treatment [41]. Other candidates under investigation include ligelizumab and quilizumab, which bind to membrane-bound IgE (mIgE) on the B cell and plasmablasts, leading to depletion [42]. 

## Challenges and gaps 

The unmet needs, and possible current and future solutions to address them, include primarily the broader and more intensive application of personalized and precision medicine approaches to patient stratification and AIT management. Significant progress has to be made in the application of more precise phenotyping and endotyping based on mHealth, CRD, and immune biomarker assessment aiming at the better prospective and retrospective stratification of patients according to their theratypes. 

The methodology for clinical research has to be improved to save resources and provide higher-quality evidence for regulatory purposes. The adaptive study design embracing the AEC-derived data, as well as the assessment of combined RCT- and RWE-derived data, is of major promise. This can only be achieved by advanced management of big data, high-throughput screenings, and the application of artificial intelligence. Further development of innovative, more effective, convenient, and safer AIT preparations together with the broader application of the combination of AIT and biologicals is the key to improving patients’ compliance by reducing therapy risk, duration, and costs. This will be of great value in overcoming the challenge of regulatory approval of novel product and methodology approaches, which can broaden the spectrum of AIT-treated patients. A continuous dialogue and collaboration between regulators, basic and translational scientists, healthcare professionals (HCPs), and patients is necessary to bring innovations to clinical practice. 

## Funding 

This study was supported by grant SUB.A020.21.018 of the Medical University in Wroclaw, Poland. 

## Conflict of interest 

MJ reports personal fees from personal fees ALK-Abello, Allergopharma, Stallergenes, Anergis, Allergy Therapeutics, Leti, HAL, GSK, Novartis, Teva, Takeda, Chiesi, Pfizer, Regeneron, Astra Zeneka, Lallemand, Shire, CELLTRION Inc. Genetech, Roche, Verona, Lek Pharmaceuticals, Arcutis Biotherapeutics, FAES FARMA outside of submitted work; and is the Deputy Editor of Allergy and past president of EAACI. MZW declares to be the EAACI Knowledge Hub Deputy Editor. 

**Table 1.** Novel biomarkers for AIT patient stratification.

**Biomarker**		**Ref**
Predictors of success measured before AIT	↑ Periostin level	[12]
	↑ Eosinophil inflammation + EAR and LAR reaction	
Predictors of success measured during AIT	↓ Basophil sensitivity	[13]
	↑ IL10^+^ CTLA4^+^ ILCs	[9]
	↑ Serum IgA, IgE, and IgG4 ↑ Nasal fluid IgA1	
	↓ HETE (metabolic biomarkers)	[14]
Predictors of AIT failure	↑ IL-6^+^ CD38^+^ B cells	[10] [15]
	↑ Allergen-specific IL-4^+^ and IL-13^+^ CD4^+^ cells (T2)	

AIT = allergen immunotherapy; B = lymphocytes B; EAR/LAR = early/late asthmatic reaction; HETE = hydroxyeicosatetraenoic acid; Ig = immunoglobulin; IL = interleukin; ILC = innate lymphoid cell; T2 = type 2 immune response.

**Table 2.** State of the art of allergen immunotherapy preparations.

**Recombinant allergens**	**Target molecules/extracts**	**Ref.**
Recombinant wildtype allergens	rBet v 1, rPhl p1/2/5a/5b/6, rMal d 1	[33] [34] [36] [37] [43] [44]
Peptide-based technology	Fel d 1, Amb a 1, Bet v 1, Ole e1, peptides from grass pollen and HDM allergens	
Nucleic acid-based	DNA plasmid encoding Cry J 2 allergen	
Recombinant hypoallergens	Bet v 1, Fel d 1, Ara h1/2/3, grass pollen allergens	
Recombinant IgG mAb against specific epitopes of allergens	Inhibition of IgE binding to allergens	
Adjuvants/carries	Target molecules/extracts	
Nanoparticles	Bet v 1, rChe a 3, Der p 1 and 2, Der f 2, PLA2, nOle e 1, Phl 5, Fel d 1, Ara h 2, Par j1 and 2, Cry j 1, Art v 1, peanut extract	[38] [45] [46] [47]
Polysaccharide polymers	Phl p 5, rFel d 1, Der f 2	
Liposomes	Par j 2, Japanese cedar pollen, HMD, cat, cockroach extract	
VLPs	Der p 1 and 2, Fel d 1, Art v 1, Ara h 1 and 2, Che a 3	
Glycodendrimers	Pru p 3, Ole e 1	
Others	Mechanism of action	
Allergoid–mannan conjugates	Reprogramed monocytes into stable tolerogenic DCs	[8]
Biologicals + AIT	Observed outcomes	
Omalizumab	Smaller risk of side-effects, increased capacity of allergen tolerance	[39] [40] [41]
Dupilumab		
Tezepelumab		

AIT = allergen immunotherapy; B = B lymphocyte; DC = dendritic cell; DNA = deoxyribonucleic acid; HDM = house dust mite; Ig = immunoglobulin; mAb = monoclonal antibody; Th2 = type 2 T-helper lymphocyte; VLP = virus-like particle.
